# Serum pancreatitis-associated protein 1 concentrations in dogs with acute signs of gastrointestinal disease and normal or abnormal DGGR lipase activity

**DOI:** 10.1093/jvimsj/aalag015

**Published:** 2026-02-23

**Authors:** Melanie Sidler, A Katrin Helfer-Hungerbuehler, Daniel Brugger, Barbara Riond, Matthias Dennler, Stefan Unterer, Peter H Kook

**Affiliations:** Clinic for Small Animal Internal Medicine, Vetsuisse Faculty, University of Zurich, Zurich 8057, Switzerland; Clinical Laboratory, Vetsuisse Faculty, University of Zurich, Zurich 8057, Switzerland; Institute for Animal Nutrition and Dietetics, Vetsuisse Faculty, University of Zurich, Zurich 8057, Switzerland; Clinical Laboratory, Vetsuisse Faculty, University of Zurich, Zurich 8057, Switzerland; Clinic of Diagnostic Imaging, Department of Clinical Services, Vetsuisse Faculty, University of Zurich, Zurich 8057, Switzerland; Clinic for Small Animal Internal Medicine, Vetsuisse Faculty, University of Zurich, Zurich 8057, Switzerland; Clinic for Small Animal Internal Medicine, Vetsuisse Faculty, University of Zurich, Zurich 8057, Switzerland

**Keywords:** PAP-1, C-reactive protein, dogs, lipase activity, MCAI, pancreatitis, pancreatic ultrasonography

## Abstract

**Background:**

Pancreatitis-associated protein (PAP-1) is synthesized during acute pancreatitis (AP) and chronic enteropathy in people. Serum PAP-1 concentration (PAP-1) has not been measured prospectively in dogs.

**Objectives:**

Evaluate whether PAP-1 differentiates suspected AP (sAP) diagnosed by abnormal DGGR-lipase activity from non-pancreatic acute gastrointestinal disease (aGId) diagnosed by normal DGGR-lipase activity.

**Animals:**

Twenty-six dogs with sAP, 48 dogs with aGId based on signs of acute gastrointestinal disease, lipase activity > 450 U/L (reference interval [RI],17-156 U/L) and maximally 20 U/L > RI, respectively. Forty healthy control dogs.

**Methods:**

Prospective daily assessment included a simplified modified canine activity index (MCAI). PAP-1, lipase activity, C-reactive protein concentration (CRP) were measured daily. PAP-1 assay validation comprised precision, interferences, linearity, and RI establishment.

**Results:**

Lower/upper PAP-1 quantification limits were 0.2 and 6.0 μg/mL, linearity was excellent (R^2^ 0.999) at high, acceptable (R^2^ 0.966) at low PAP-1 concentrations. Intra-, inter-run precision was ≤5%, ≤22%, PAP-1 remained stable for 15 days (room temperature), no interferences were found. Duration of hospitalization and clinical disease severity did not differ between groups. At admission, PAP-1 above RI in 50% and 48 % of sAP and aGId dogs, respectively (sAP median, range 1.88 μg/mL, 0.2-6.0 vs. aGId 1.57 μg/mL, 0.2-6.0; RI, <1.9 μg/mL). PAP-1 did not differ significantly between groups irrespective of observation time points. PAP-1 correlated significantly with CRP in sAP (*r*_s_ = 0.623) and aGId (*r*_s_ = 0.483). PAP-1 correlated significantly with lipase activity (*r*_s_ = 0.474) in sAP, with MCAI (*r*_s_ = 0.342) in aGId.

**Conclusion and clinical importance:**

Serum PAP-1 reflects inflammation rather than underlying disease processes, and does not differentiate sAP from aGId.

## Introduction

Acute pancreatitis (AP) is a common clinical diagnosis in dogs. Serum lipase, measured either as an activity (1,2-o-dilauryl-rac-glycero-3-glutaric acid-(6′-methylresorufin) ester [DDGR]-based catalytic lipase assays)^1^ or a concentration (pancreatic lipase immunoreactivity [PLI]),^2^ is widely regarded as the laboratory biomarker of choice for a clinical diagnosis of AP. Both tests correlate strongly with each other.^1^^,^^3–11^ Both lipase assays [(Roche (DGGR-based) lipase activity^1^ and Spec PL (PLI)] yield essentially the same results and have equivalent clinical relevance when lipase is measured daily during hospitalization in dogs with AP^4^ as well as during elective re-checks.^11^ C-reactive protein concentration (CRP) is the most important acute phase protein in dogs, and measured in dogs with AP to assess the extent of systemic inflammation. Pancreatic lipase release and systemic inflammation do not run in parallel.^4^^,^^12^ CRP has not been compared between AP and acute gastrointestinal disease (aGId) when measured over time during hospitalization.

In humans, serum pancreatitis-associated protein 1 (PAP-1), a 17 kDa secretory protein undetectable or minimally expressed under physiological conditions but synthesized during AP in pancreatic acinar cells, is suggested as a useful biomarker for AP.^13^ It is a member of the regenerating islet-derived protein (REG) group consisting of C-type lectin like proteins.^14^^,^^15^  *REG3A* is the official gene name in humans, but PAP-1 is used especially in the context of inflammation and pancreatitis.^14^ Serum PAP-1 concentrations are valuable when assessing disease severity and recovery process in people with AP.^16^^,^^17^ Inhibition of PAP-1 in experimental models of AP exacerbates necrosis and neutrophil infiltration in pancreatic tissue.^18^ Serum PAP-1 concentration is also abnormally high in humans with chronic inflammatory bowel disease such as Crohn’s disease^19–23^ and ulcerative colitis^21^^,^^22^ but within reference intervals (RI) in people with acute enteritis.^21^ High fecal PAP-1 concentrations are also reported in cats and dogs with chronic enteropathies.^24^^,^^25^ Additionally, there is increased expression of a REG3 subfamily protein member in dogs,^26^ and a REG3 protein member (a homolog of human REG3A also known as PAP-1) is higher in plasma from dogs with suspected AP.^27^ So far, PAP-1 has not been measured prospectively in serum of dogs.

Therefore we aimed to (1) measure serum PAP-1 concentrations in dogs hospitalized for suspected AP (sAP) as well as in dogs with aGId daily during hospitalization and compare results between groups, (2) determine if associations exist between PAP-1 concentration and ultrasonographic pancreatic findings and to describe associations between concentrations of PAP-1, CRP, lipase activity, and clinical severity over time during hospitalization. Our hypotheses were: (1) serum PAP-1 concentration in dogs is increased in AP but not in aGId, and (2) serum PAP-1 concentration correlates with clinical severity during hospitalization in dogs with AP.

## Materials and methods

### Study cohort

Dogs with sAP or aGId presenting to the Clinic for Small Animal Internal Medicine of the Vetsuisse faculty, University of Zurich, Switzerland between April 2023 and March 2024 were considered for this prospective study. Inclusion criteria for sAP dogs were acute onset (< 7 days) of ≥2 clinical signs suggestive of AP including vomiting, abdominal pain, diarrhea, lethargy, and inappetence, DGGR-lipase activity^1^ >450 U/L (LIPC, Roche on Cobas, Roche Diagnostics, Rotkreuz, Switzerland, RI, 17-156 U/L), and absence of extra-pancreatic diseases that could also explain clinical signs and hyperlipasemia (eg, intestinal foreign bodies^28^). The aGId group consisted of dogs with acute onset (<7 days) of aforementioned clinical signs, and a lipase activity within 20 U/L of the upper RI. The current LIPC Roche lipase activity RI [17 U/L (90% CI 12-21) − 156 (90% CI 129-224) U/L] is higher than our previous RI (24-108 U/L).^1^

Dogs with major comorbidities (eg, intestinal obstruction, abdominal mass, cardiopulmonary disease, liver failure, metabolic disease, autoimmune disease, systemic infection, neoplastic disease, suspicion of endocrine disease, acute kidney injury, or chronic kidney disease IRIS stage >2) and dogs with diagnoses of chronic enteropathy based on clinical or histological biopsy data were excluded. Dogs with samples with signs of interference (hemolysis or icterus) based on routine indices were also excluded.

For calculation of a PAP-1 RI, blood samples were collected from 40 clinically healthy staff- or student-owned dogs with a lipase activity within RI. All 40 dogs had no abnormalities detected on physical examination, no history of gastrointestinal disease, and were receiving no medication except regular ecto-/endoparasite prophylaxis.

The study was approved by the Cantonal Veterinary office of Zurich and conducted in accordance with guidelines established by the Animal Welfare Act of Switzerland (No. ZH029/2023).

### Study design and blood sample collection

Once eligible cases were identified, dog owners were contacted by the study investigator for voluntary study participation. After owner consent documentation, standardized medical histories were obtained including duration of disease prior to presentation in hours. Presence of appetite, vomiting, diarrhea, abdominal pain, dehydration, lethargy, and blood in feces were assessed and graded using a slightly simplified modified canine activity index (MCAI).^12^ Minor modifications were made for assessments of appetite, abdominal pain, and fecal consistency (appetite graded as normal (=0), decreased (=1), absent (=2); abdominal pain graded as none (=0), suspicion of abdominal pain (=1), abdominal pain (=2); fecal consistency graded as normal (=0), soft or poorly formed (=1), or watery diarrhea (=2)). CRP was measured using a turbidimetric immunoassay (Gentian AS, Moss, Norway, RI, 0-10.2 mg/L). MCAI, PAP-1, lipase activity, and CRP were measured daily during hospitalization.

With the exception of one examination performed by a board-certified internist experienced in abdominal ultrasonography, abdominal ultrasonographic examinations (LOGIQ E10, GE Healthcare) were performed by board-certified radiologists or residents-in-training under direct supervision of a board-certified radiologist. Ultrasonographic pancreatic appearances were assessed using a slightly modified ultrasonographic pancreatic assessment severity score (UPASS).^29^ Mesenteric echogenicity was graded as either normal (=0) or hyperechoic (=1).

### Evaluation of the SPARCL PAP-1 immunoassay

M&M of the SPARCL PAP-1 assay validation can be found in the Appendix.

### Statistical analyses

For each outcome variable (PAP-1, CRP, and lipase activity) we fitted a separate generalized linear mixed model with a Gamma error distribution and log-link function in PROC GLIMMIX (SAS 9.4). For admission values, fixed effects were treatment group and sex (no interaction), with a random intercept for “dog”; parameters were estimated by maximum likelihood with the Laplace approximation. For longitudinal analyses, we pre-specified a small set of candidate GLMMs: (1) a base model with fixed effects for group and time, a random intercept for dog, and an Ante-dependence covariance structure of order 1 (Ante(1)) for repeated measures; (2) the same model with a group × time interaction; (3) each of the above with sex included as a covariate.

Models were compared using AICc and, for nested specifications, likelihood-ratio tests (α = 0.05; Kenward–Roger degrees-of-freedom adjustment). The final model was the most biologically plausible model with the lowest AICc; when ΔAICc <2, we preferred the more parsimonious specification. Rationale for sex: sex was included a priori as a potential confounder (biological plausibility for inflammatory differences and possible imbalance across groups). No further covariates were considered given the sample size to avoid overfitting. Model specifications followed the approach described above; full output tables and sensitivity analyses are provided in the Supplementary material.

Multiple-means comparisons in GLIMMIX used the Tukey–Kramer procedure, which controls the family-wise error rate by comparing all pairwise mean differences to a single critical value from the studentized-range distribution. Spearman’s rank correlation coefficients assessed associations among PAP-1, CRP, lipase, and with MCAI. Ordinal clinical scores were analyzed at each time-point with 2-sided Mann–Whitney U tests, with Bonferroni adjustment across time-points (Bonferroni Adjustment: *P* ≤ .008). Mann–Whitney U tests also compared (1) admission PAP-1 among sAP, aGId, and healthy controls (Bonferroni Adjustment: *P* ≤ .017); (2) PAP-1 between dogs with vs without individual pancreatic ultrasonographic findings; and (3) total MCAI, admission PAP-1 and CRP, and UPASS between sAP dogs with persistently high (>450 U/L) vs rapidly decreasing (<450 U/L on day 2) lipase activity. Type I error threshold was *P* ≤ .05 if not stated otherwise above.

RIs were calculated in Analyze-it for Microsoft Excel version 2018 (Build 14326.20404) using a non-parametric bootstrap approach, reporting the 2.5th and 97.5th percentiles with 90% confidence intervals. Details and full results of the post-hoc power analyses are provided in Supplementary files.

## Results

### Dogs

Twenty-six dogs with sAP and 48 with aGId were included. Signalment is shown in Table 1. Median (range) duration of disease was 36 h (3-96) in AP and 48 h (3-168) in aGId dogs.

**Table 1:** Signalment of sAP, aGId, and healthy control dogs.

		sAP dogs (*n* = 26)	aGId dogs (*n* = 48)	Healthy dogs (*n* = 40)
**Sex**	*n* (%)	11 female (42) 15 male (58)	20 female (42) 28 male (58)	26 females (65) 14 males (35)
**Age (years)**	Median (range)	7.4 (0.4-14.4)	4.9 (0.6-11.8)	5.2 (0.7-11.9)
**Weight (kg)**	Median (range)	6.4 (1.2-38.0)	10.5 (2.0-49.7)	19.9 (1.5-62.0)
**Breed**	(n)	Mixed breed (4), Chihuahua (4), German Spitz (3), Poodle (3), Jack Russel Terrier (2), Australian Cobberdog (1), Bernese Mountain Dog (1), Bolonka (1), Cocker Spaniel (1), Dachshund (1), Flat Coated Retriever (1), Greyhound (1), Labrador Retriever (1), Shetland Sheepdog (1), Welsh Terrier (1)	Dachshund (7), Mixed breed (5), German Spitz (3), Labrador Retriever (3), Miniature Schnauzer (3), Poodle (3), Havanese (2), Magyar Viszla (2), Australian Shephard (1), Barsoi (1), Basset Hound (1), Beagle (1), Bolonka (1), Border Terrier (1), Entlebuch Mountain Dog (1), Flat Coated Retriever (1), German Shephard (1), Goldendoodle (1), Lagotto Romagnolo (1), Maltese (1), Podenco Canario (1), Prague Rattler (1), Pug (1), Rhodesian Ridgeback (1), Serbian Hound (1), Weimaraner (1), Welsh Corgi Pembroke (1), Yorkshire Terrier (1)	Mixed breed (21), Labrador Retriever (3), German Pointer (2), Australien Kelpie (1), Bearded Coliie (1), Border Collie (1), Great Dane (1), Kooikerhondje (1), Yorkshire Terrier (1), Labradoodle (1), Australien Cattle Dog (1), Australien Shephard (1), Whippet (1), Icelandic Sheepdog (1), German Shephard (1), Tibetan Terrier (1), Eurasien (1)

### Evaluation of PAP-1 measurement using the Vetbio-1 SPARCL assay

Results of the SPARCL PAP-1 assay validation can be found in the Appendix.

### Serum PAP-1 at admission

**Figure 1:**
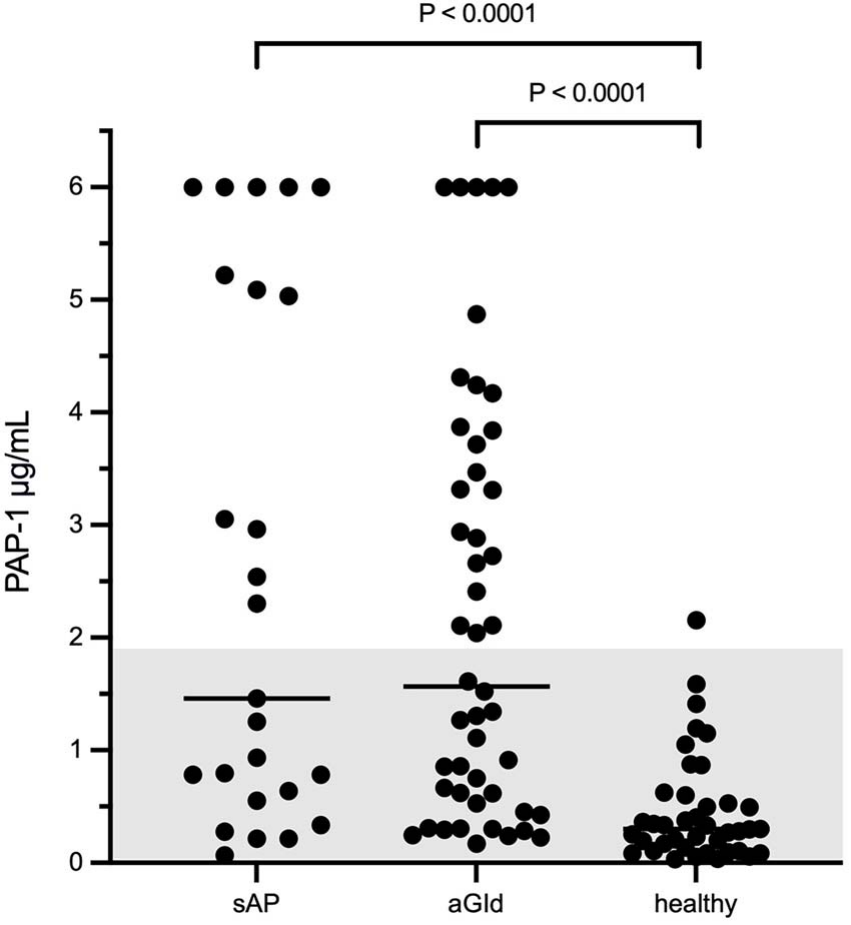
Serum PAP-1 concentration at admission was significantly higher in both sAP (*n* = 26) and aGId (*n* = 48) compared to healthy control dogs (*n* = 40). Mann-Whitney U test, an alpha level of 0.05 was used to determine statistical significance. When the Bonferroni correction was applied, an adjusted significance level of 0.017 was calculated. The gray-shaded area represents the RI. Please note that plotted points can be indistinguishable because of individual values that are too close or identical.

Serum PAP-1 concentration of sAP and aGId at admission, as well as healthy control dogs are shown in Figure 1. Both groups did not differ from each other, while both sAP (*P* < .0001) and aGId dogs (*P* < .0001) had significantly higher serum PAP-1 concentration compared to healthy control dogs.

#### Progression of serum PAP-1, lipase activity, and CRP during hospitalization

**Figure 2:**
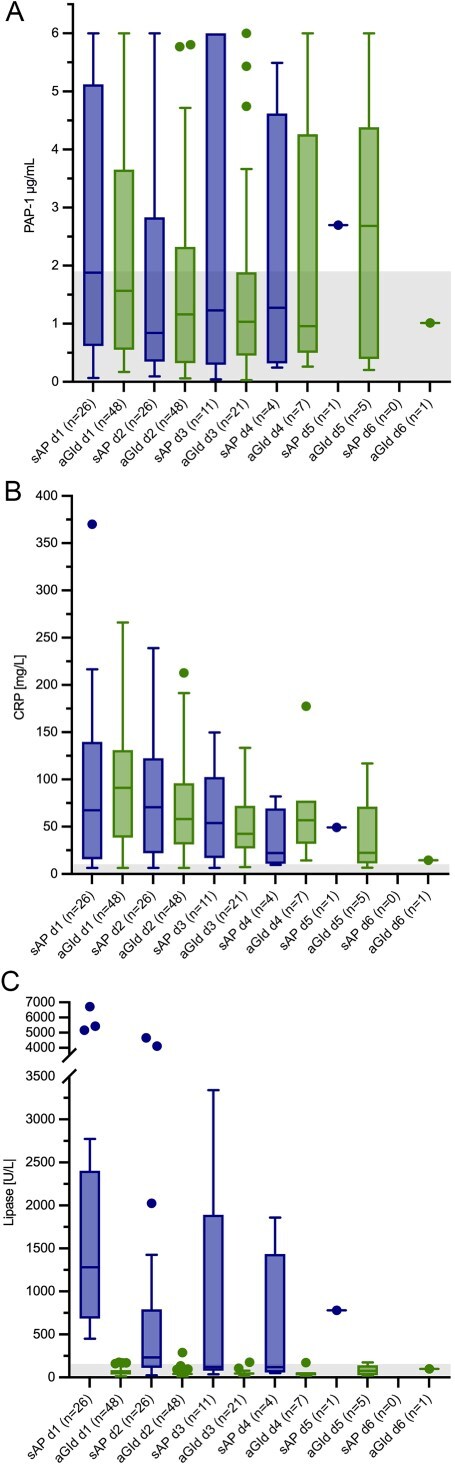
(A) Progression of serum PAP-1 concentration over time in 26 and 48 hospitalized dogs with sAP and aGId, respectively. The course of PAP-1 concentration did not significantly differ between groups. General linear mixed model, an alpha level of 0.05 was used to determine statistical significance. The gray-shaded area represents the RI. (B) Progression of serum CRP concentration over time in 26 and 48 hospitalized dogs with sAP and aGId, respectively. The course of CRP concentration did not significantly differ between groups. General linear mixed model, an alpha level of 0.05 was used to determine statistical significance. The gray-shaded area represents the RI. (C) Progression of serum lipase activity over time in 26 and 48 hospitalized dogs with sAP and aGId, respectively. The gray-shaded area represents the RI. Please note that plotted points can be indistinguishable because of individual values that are too close or identical.

Irrespective of time points of observation serum PAP-1 concentration did not differ significantly between sAP and aGId group (*P* = .08). Progression of PAP-1, CRP, and lipase activity is shown in Figure 2A-C. Median (range) values of PAP-1, CRP, and lipase activity of AP and aGId dogs during hospitalization are shown in Table 2.

**Table 2:** Median (range) results of PAP, CRP, and lipase activity during hospitalization in dogs with AP and aGId.

Group	Analyte	Day 1	Day 2	Day 3	Day 4	Day 5	Day 6
**AP**	**PAP (μg/mL)**	1.88 (0.2-6.0)	0.84 (0.2-6.0)	1.23 (0.2-6.0)	1.28 (0.2-5.49)	2.7	
	**CRP (mg/L)**	67.5 (6.4-370)	70.5 (6.4-239)	53.9 (6.4-149.8)	22.2 (9.7-82)	49.3	
	**Lipase (U/L)**	1280 (452-6712)	232.5 (24-4656)	122 (37-3340)	120.5 (51-1859)	780	
	***n* dogs hospitalized**	26	26	11	4	1	0
**aGId**	**PAP (μg/mL)**	1.57 (0.2-6.0)	1.16 (0.2-5.8)	1.03 (0.2-6.0)	0.96 (0.26-6.0)	2.68 (0.2-6.0)	1.01
	**CRP (mg/L)**	91.15 (6.4-239)	58 (6.5-212.8)	42.5 (7.2-133.4)	56.8 (14.2-177.5)	22.3 (6.7-116.9)	14.5
	**Lipase (U/L)**	49.5 (14-176)	40.5 (14-289)	45 (17-176)	49 (19-173)	76 (22-175)	99
	***n* dogs hospitalized**	48	48	21	7	5	1

At admission, 13/26 sAP (50%) and 23/48 aGId (48%) dogs had serum PAP-1 concentrations above RI (Table 3). During hospitalization, PAP-1 concentration increased > RI in only 1/13 sAP dogs that had d1 PAP-1 within RI. In the aGId group, PAP-1 concentration increased > RI in 5/25 dogs with d1 PAP-1 within RI.

**Table 3:** Number (%) of AP and aGId cases above reference interval of PAP, CRP, and lipase activity.

Group	Analyte	RI		Day 1	Day 2	Day 3	Day 4	Day 5	Day 6
**AP**	**PAP**	<1.9 μg/mL	>1.9 μg/mL; *n* (%)	13 (50)	10 (38)	4 (36)	2 (50)	1 (100)	
	**CRP**	≤10.2 mg/L	>10.2 mg/L; *n* (%)	21 (81)	21 (81)	9 (82)	3 (75)	1 (100)	
	**Lipase**	17-156 U/L	>156 U/L; *n* (%)	26 (100)	16 (62)	4 (36)	2 (50)	1 (100)	
	***n* dogs hospitalized**			26	26	11	4	1	0
**aGId**	**PAP**	<1.9 μg/mL	>1.9 μg/mL; *n* (%)	23 (48)	17 (35)	5 (24)	3 (43)	3 (60)	0 (0)
	**CRP**	≤10.2 mg/L	>10.2 mg/L; *n* (%)	45 (94)	45 (94)	20 (95)	7 (100)	4 (80)	1 (100)
	**Lipase**	17-156 U/L	>156 U/L; *n* (%)	4 (8)	1 (2)	1 (5)	1 (14)	1 (20)	0 (0)
	***n* dogs hospitalized**			48	48	21	7	5	1

Increased CRP was found in 21/26 sAP (81%) and 45/48 aGId (94%) dogs at day 1 (Table 3). Irrespective of time points of observation CRP did not differ significantly between AP and aGId dogs (*P* = .6).

### PAP-1 concentrations above the upper limit of quantification in sAP dogs

In total, 5/26 (19%) dogs had PAP-1 > upper limit of quantification (ULQ) of 6.0 μg/mL. One dog had a concentration >6.0 μg/mL only at admission, while 4 dogs had PAP-1 exceeding 6.0 μg/mL throughout hospitalization. These 4 dogs had significantly higher admission CRP (median, 211.4 mg/L, range, 125.1-370.0, *P* = .002) as well as lipase activities at day 2 (median, 2655 U/L, range, 632-4656, *P* = .004) compared to the other sAP dogs (median CRP day 1, 53.8 mg/L, range, 6.4-171.8; median lipase activity day 2, 205 U/L, range, 24-2025). At day 3, 3 of these 4 dogs were still hospitalized and had significantly higher day 3 lipase activities (median, 2921 U/L, range, 1891-3340; *P* = 0.01) compared to the other sAP dogs at day 3 (*n* = 8) (median, 98 U/L, range, 37-1066). UPASS, and frequency of individual pancreatic ultrasonographic abnormalities were not significantly different between sAP dogs with PAP-1 concentrations consistently > ULQ and the other sAP dogs.

### PAP-1 concentrations above ULQ in aGId dogs

In total 6/48 (13%) dogs had PAP-1 concentrations >6.0 μg/mL. Three dogs had concentrations >6.0 μg/mL only at admission (their CRP concentrations were 149.8, 113.3, and 266.2 mg/L). While one dog had PAP-1 values > ULQ on three consecutive days (day 3-day 5), corresponding CRP was 68.4, 77.5, and 116.9 mg/L.

### Progression of lipase activity and relationship with PAP-1 in sAP dogs during hospitalization

**Figure 3:**
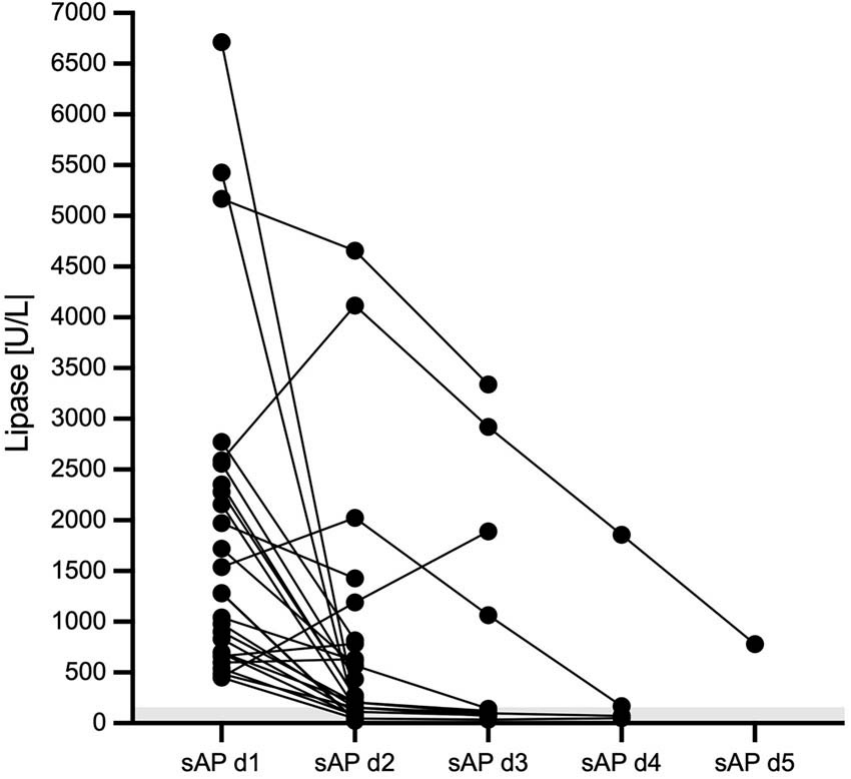
Progression of serum lipase activity over time in 26 hospitalized dogs with sAP. The gray-shaded area represents the RI. Please note that plotted points can be indistinguishable because of individual values that are too close or identical.

Progression of lipase activity in sAP dogs during hospitalization is shown in Figure 3. In 21/26 dogs (81%) lipase activity decreased from day 1 to day 2, but remained > RI in 16/26 dogs (62%) on day 2, ranging from 204-4656 U/L. Lipase activity further increased in 5/26 dogs (19%) from day 1 to day 2 (from 452 to 1192, from 599 to 632, from 2591 to 4117, from 1539 to 2025, and from 661 to 785 U/L). The first three of these 5 dogs with further increasing lipase activities had PAP-1 concentrations > ULQ of 6.0 μg/mL on both day 1 and day 2, PAP-1 increased from 1.46 on day 1 to 2.58 μg/mL on day 2 in the 4th dog, while PAP-1 was within RI at the first two hospitalization days in the 5th dog. On day 3, lipase activity remained increased in 4/11 dogs (36%) (range, 1066-3340 U/L) still hospitalized at that time. While lipase activity decreased from day 2 to day 3 in 10/11 dogs (91%), it increased in 1 dog (9%) from 1192 on day 2 to 1891 U/L on day 3. The latter dog had PAP-1 concentrations > 6.0 μg/mL on both day 2 and day 3. On day 4, lipase activity was still > RI in 2/4 (50%) dogs still hospitalized (167 and 1859 U/L). Both dogs had PAP-1 concentrations > RI (2.02, 5.49 μg/mL).

#### Progression of lipase activity and relationship with PAP-1 in aGId dogs during hospitalization

**Figure 4:**
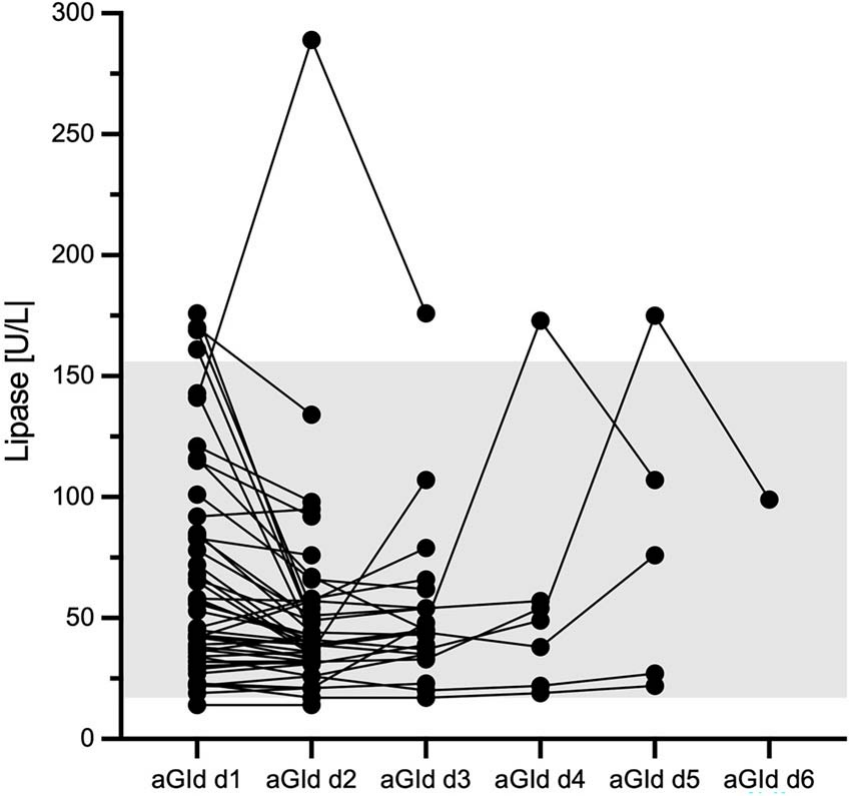
Progression of serum lipase activity over time in 48 hospitalized dogs with aGId. The gray-shaded area represents the RI. Please note that plotted points can beindistinguishable because of individual values that are too close or identical.

Progression of lipase activity in aGId dogs during hospitalization is shown in Figure 4. Lipase activity increased >176 U/L during hospitalization in only one (2%) dog. Progression of lipase activity in this dog was 141 (day 1), 289 (day 2), and 176 U/L (day 3; day of discharge). Corresponding PAP-1 concentrations were all within RI (0.3 day 1, 0.29 day 2, and 0.2 μg/mL day 3). Pancreatic ultrasonography was unremarkable in this dog.

Two other aGId dogs had serum lipase > RI but <176 U/L once during hospitalization (173, 175 U/L). Respective PAP-1 concentrations were 4.24 (day 1), 3.62 (day 2), 6.0 (day 3), 6.0 (day 4), and 6.0 (day 5) as well as 6.0 (day 1), 5.77 (day 2), 1.07 (day 3), 0.5 (day 4), 2.68 (day 5), and 1.01 μg/mL (day 6). Ultrasonography of the pancreas was also normal in both dogs.

### Results of sAP dogs with lipase activity consistently >450 U/L

Eleven (42%) sAP dogs had lipase activities >450 U/L during the first 2 days of hospitalization, remaining above the inclusion criterion of approximately 3 times the upper RI limit (Figure S1). These dogs had significantly higher PAP-1 and CRP at admission (median PAP-1, 5.03 μg/mL, range, 0.55-6.0, *P* = .03; median CRP, 125.1 mg/L, range, 13.3-370.0, *P* = .04), as well as UPASS (Figure S2) (median UPASS, 5, range 2-6, *P* = .01) compared to 15 sAP dogs with rapidly decreasing lipase activities (median PAP-1, 0.93 μg/mL, range, 0.2-5.22; median CRP, 36.4 mg/L, range, 6.4-160.5; median UPASS, 3, range, 1-5). The course of PAP-1 and CRP during hospitalization in these 11 AP dogs is shown in Figures S3 and S4. Moreover, PAP-1 concentrations at day 2 were significantly higher and total MCAI at d2 significantly lower in the 11 sAP dogs with consistently increased lipase activity >450 U/L during hospitalization (median PAP-1 d2, 2.58 μg/mL, range, 0.36-6.0, *P* = .004; median MCAI day 2, 1, range, 0-4, *P* = .0004) in comparison to sAP dogs with rapidly decreasing lipase activities (median PAP-1 day 2, 0.53 μg/mL, range, 0.2-4.67; median MCAI day 2, day 3, range, 1-6). At day 3, 5 of these 11 dogs were still hospitalized and had significantly higher day 3 PAP-1 (median PAP-1 day 3, 6.0 μg/mL, range, 1.23-6.0, *P* = .02) compared to the other sAP dogs (*n* = 6) (median PAP-1 day 3, 0.33 μg/mL, range, 0.2-4.1). Frequency of individual abnormal pancreatic ultrasonographic variables did not significantly differ between sAP dogs with consistently increased lipase activity >450 U/L during hospitalization and without.

### Correlations between PAP-1 and lipase activity, CRP, and MCAI over time

Within the sAP group, PAP-1 correlated moderately with both lipase activity (*r*_s_ = 0.474, *P* < .0001) and CRP (*r*_s_ = 0.623, *P* < .0001) irrespective of time points of observation but not with total MCAI (Table 4). Within the aGId group, PAP-1 correlated moderately with both CRP (*r*_s_ = 0.483, *P* < .0001) and total MCAI (*r*_s_ = 0.342, *P* < .0001) irrespective of time points of observation but not with lipase activity (Table 4).

**Table 4:** Spearman’s rank correlation coefficient (*r*_s_) and statistical significance (*P*-value) for the correlations between PAP and lipase activity, CRP, and total MCAI over time.

Group	PAP with lipase	PAP with CRP	PAP with total MCAI	Lipase with CRP	Lipase with total MCAI	CRP with total MCAI
**sAP**	*r*_s_ = 0.474 *P* < .0001^a^	*r*_s_ = 0.623 *P* < .0001^a^	*r*_s_ = −0.096 *P* = .42	*r*_s_ = 0.159 *P* = .18	*r*_s_ = −0.057 *P* = .63	*r*_s_ = 0.029 *P* = .8
**aGId**	*r*_s_ = −0.014 *P* = .87	*r*_s_ = 0.483 *P* < .0001^a^	*r*_s_ = 0.342 *P* < .0001^a^	*r*_s_ = 0.018 *P* = .84	*r*_s_ = 0.036 *P* = .68	*r*_s_ = 0.398 *P* < .0001^a^

An alpha level of 0.05 was used to determine statistical significance.

a Statistically significant value.

### Ultrasonographic findings and associations of PAP-1 with pancreatic ultrasonographic changes

In all dogs with sAP and in 44/48 (92%) dogs with aGId ultrasonography was performed. All sAP dogs had ultrasonographic evidence of pancreatopathy (17 dogs with specification “acute,” 6 dogs with “acute on chronic,” and 3 with “chronic”), 15 dogs (58%) had an enlarged, and 16 (62%) a hypoechoic pancreas.

In the aGId group, 12/44 (27%) dogs had ultrasonographic evidence of pancreatic disease, 3 dogs (7%) had a hypoechoic pancreas.

PAP-1 did not correlate significantly with UPASS in both groups. AP dogs with an enlarged pancreas (*n* = 15) had significantly higher PAP-1 concentrations (median 3.4 μg/mL; range, 0.33-6.0) compared to a median of 0.78 μg/mL (range, 0.2-6.0) in sAP dogs without pancreatic enlargement (*n* = 11) (*P* = .02). Furthermore, sAP dogs with peripancreatic free fluid (*n* = 3) had significantly higher PAP-1 (median 6.0 μg/mL, range 5.03-6.0) in comparison to those without (*n* = 23) (median 1.26 μg/mL, range 0.2-6.0) (*P* = .03). PAP-1 was not significantly different in the sAP group when a hypoechoic, hyperechoic, mixed-echoic pancreas, or a hyperechoic mesentery were present or absent.

In aGId, PAP-1 did not differ in dogs with a hypoechoic pancreas compared to those without. All dogs with a hypoechoic pancreas had lipase activities <RI both at admission and throughout hospitalization.

### Clinical findings during hospitalization

**Figure 5:**
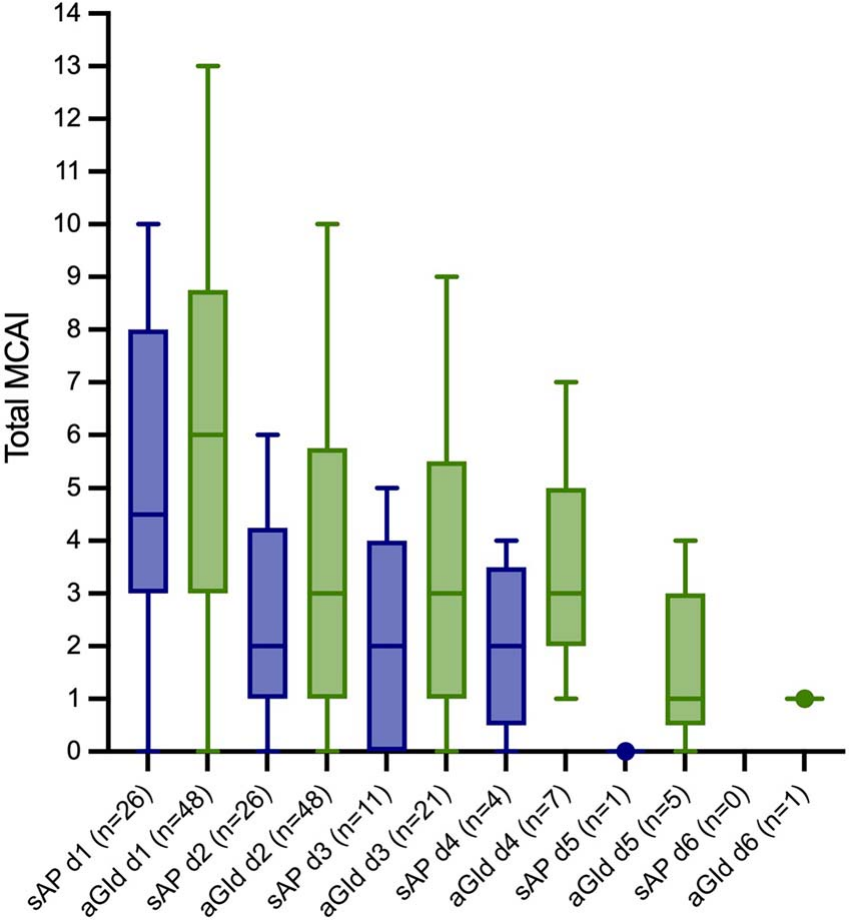
The course of the total modified canine activity index (MCAI) did not significantly differ between dogs with sAP and aGId. Mann-Whitney U tests, an alpha level of 0.05630 was used to determine statistical significance. When the Bonferroni correction was applied, an adjusted significance level of 0.008 was calculated.

Total MCAI (Figure 5) as well as individual MCAI components did not differ significantly between sAP and aGId on days 1-4. Fecal consistency was worse in aGId compared to sAP; however, the *P*-value did not reach statistical significance (*P* = .052). SAP and aGId dogs were hospitalized for a median (range) of 3 (1-5) and 2.5 days (1-5), respectively. In the sAP group, 25/26 (96%) dogs were discharged from the hospital. One dog died unexpectedly during hospitalization (day 3). All dogs with aGId were discharged from hospital. All discharged dogs were alive at day 7 post discharge (re-check at our hospital or telephone re-check). PAP-1 at admission correlated moderately with duration of hospitalization in sAP dogs (r_s_ = 0.422, *P* = .04) but not in aGId dogs.

## Discussion

In this study, we describe the daily course of serum PAP-1 in dogs hospitalized for sAP and aGId. Serum PAP-1 levels were significantly higher in dogs with both sAP and aGId relative to healthy controls. Almost half of dogs in both groups had concentrations within calculated RI. Although PAP-1 concentrations were significantly higher in a subset of sAP dogs with evidence of more severe disease (persistently abnormal lipase activities during hospitalization, ultrasonographic evidence of an enlarged pancreas) PAP-1 levels were neither significantly different at admission nor during any day of hospitalization between both groups. While PAP-1 could not distinguish dogs with sAP from dogs with aGId, PAP-1 values correlated with CRP in both groups (Table 4).

In humans, PAP-1 is a reliable indicator for disease severity and healing process of AP.^16^^,^^17^ In particular, continuously abnormal serum PAP-1 levels are more marked in patients with severe disease.^16^ Other studies suggested that PAP-1 might not be a useful serum marker for the diagnosis of AP.^30^^,^^31^ Determination of serum PAP-1 concentration only at admission is not clinically useful due to overlapping ranges between patients with AP and those with non-pancreatic acute abdomen.^17^^,^^30^^,^^31^ Our findings in dogs go in the same direction, also with regard to PAP-1 measurements over time. Serum PAP-1 in people with AP began to increase on day 2 or 3, peaked on day 7, then normalized with recovery from AP.^30^ This was in contrast to serum lipase activity, which peaked on day 1 and then rapidly decreased.^30^ In another study, peak concentrations of serum PAP-1 occurred even later up to 13 days depending on severity of AP.^16^ Given the shorter hospitalization in our study, we cannot exclude that further PAP-1 increases might have occurred after discharge in sAP dogs; however, the lack of continuous daily PAP-1 concentration increases in sAP dogs does not support this theory.

In the only study that assessed serum PAP-1 in people with acute enteritis, it was not increased,^21^ and this prompted us to measure PAP-1 in dogs with sAP compared to aGId. However, serum PAP-1 is synthesized in colonic epithelial cells in active inflammatory bowel disease in humans^21^ and PAP-1 correlates with both clinical^19^^,^^21^ and endoscopic disease severity^21^ in people with chronic enteropathies. In our study, only dogs with acute disease were included and clinical severity (MCAI) correlated significantly with serum PAP-1 in aGId dogs, although correlation was only moderate. Our results indicate that PAP-1 is also synthesized in the intestines in dogs with aGId. This was also demonstrated in a recently published study where PAP-1 was increased in dogs with acute GI disease compared to healthy controls.^27^ In that study, PAP-1 values of dogs with AP were even higher than those of dogs with aGId, which might have been due to time points of blood sampling, as it was not specified for how long dogs had already been ill. Another explanation might have been the higher clinical severity of AP dogs in that study,^27^ as there was a much higher case fatality rate among AP dogs (31%) compared to our results (4%).

We cannot exclude that some aGId dogs presented with an acute exacerbation of a preexisting chronic intestinal disease and this might have also contributed to serum PAP-1 increases. Abnormally high fecal PAP-1 concentrations are reported by the assay manufacturer in dogs with chronic enteropathy compared to healthy controls,^32^ although no information was given about the clinical condition of dogs when feces were sampled. But this appears to be less relevant, as PAP-1 is lower in dogs with chronic enteropathy than in those with acute enteritis.^27^ In contrast to aGId, serum PAP-1 did not correlate with MCAI in sAP dogs which is in contrast to findings in people with AP.^16^^,^^17^ When looking at how the MCAI score is composed, it becomes clear that dogs with additional diarrhea tend to get higher scores. If PAP-1 is also synthesized in canine intestinal epithelial cells, then it is quite likely that more severe intestinal disease is associated with higher PAP-1 concentrations.

Serum CRP and PAP-1 concentrations correlated significantly in both groups. It is proposed that PAP-1 behaves like an anti-inflammatory protein.^18^^,^^33^^,^^34^ Fecal PAP-1 concentrations are also considered to represent an acute phase protein in cats with chronic enteropathies.^24^ The anti-inflammatory function of PAP-1 seems to be mediated through modulation of signaling pathways in epithelial cells including inhibition of the NFκ-B-activation.^34^

Although PAP-1 did not correlate with UPASS, which serves as an ultrasonographic severity score independent of the final diagnosis of the radiologist, it was significantly higher when an enlarged pancreas was present and also higher in the few cases where peripancreatic fluid was present. This could mean that serum PAP-1 is higher in more severe AP cases. However, it is still unclear at what point radiologists consider a pancreas to be enlarged. The boomerang shape of the canine pancreas makes standardized measurements difficult and, in addition, the size of the pancreas can depend on the size of the dog.^35^

The present study provides insight into the daily progression of clinical disease severity in hospitalized dogs with suspected AP compared to dogs with aGId. Total clinical disease severity and CRP did not differ between groups suggesting that both, the clinical course and severity of inflammation of sAP is not much different to dogs with aGId.

This study evaluates a SPARCL assay and the VetBio-1 analyzer for their suitability to reliably measure serum PAP-1 concentrations in dogs. Good intra-run (<5%) and moderate inter-run precision values (<22%) samples were found. We currently do not know what causes this higher inter-run precision. In a previous study, we had identified high background chemiluminescence signals as an obstacle to the appropriate measurement of serum alpha-1 acid glycoprotein concentrations in cats (same SPARCL technology),^36^ so all sample preparation involving the borosilicate tubes, incubation and measurement were performed in a dark room. We think it is important that clinicians are aware of this data, because for patient monitoring, this inter-assay imprecision must be considered as it indicates considerable variability between different test runs. Another explanation could be differences in pipetting techniques between laboratory technicians. The reduced linearity observed at lower concentrations and higher dilutions remains a limitation. However, the overall response remained linear across the tested range. No indication of sigmoid behavior or signal saturation was observed at high dilutions, supporting the reliability of the assay within the clinically relevant concentration range. Considering that these values are below the upper RI (<1.9 μg/mL), and especially if high PAP-1 levels prove to be clinically relevant, this deviation is likely acceptable. Because of the rather large inter-run CV, ideally multiple patient samples over time are measured within one measurement series to improve interpretation of results and to detect smaller changes as the intra-run CV was low. Relevant inferences of hemolysis, lipemia, or hyperbilirubinemia, were absent. Regarding stability, PAP-1 was shown to be stable at room temperature for at least 15d. This suggests its suitability for clinical settings, where samples often need to be transported to the laboratory and are not stored at −80 °C.

We used lipase activity and clinical signs at admission for group assignment, and dogs did not have to have ultrasonographic features of AP. The approach is similar in people where 2 of 3 features (clinical sign “abdominal pain,” increased lipase activity, compatible imaging findings) have to be present for a clinical diagnosis of AP.^37^ We used a lipase activity cutoff of approximately 3 times the upper RI limit, a standard approach used to diagnose AP in both humans and dogs.^37–39^ A similar principle of exceeding the upper RI limit was likely applied when establishing the “consistent with pancreatitis” threshold of 400 μL for the PLI assay.^4^ The different timeline of abnormal lipase and ultrasonographic findings in the early progression of pancreatic inflammation raised our concern of potentially missing sAP cases if we had applied both inclusion criteria: increased lipase activity and at least 2 ultrasonographic features suggestive of AP. Abdominal ultrasonographic findings indicative of AP can appear as late as 2-3 days after hospital admission or remain absent in a subset of dogs with a clinical suspicion of AP and no other disease that could explain clinical signs.^40^^,^^41^ All dogs in this study were acutely ill and the duration of disease before presentation is the shortest published so far for AP (and aGId).^4^^,^^42^^,^^43^ Because we aimed to also compare PAP-1 with pancreatic ultrasonographic findings in both groups, we decided to use abnormal serum lipase activity as the distinguishing criterion. During subsequent hospitalization, lipase activity increased in only 1/48 aGId dogs (2%) above our inclusion criterion and this single increase remained below 2 × upper RI limit. The observation that dogs with acute gastrointestinal clinical signs and normal lipase at first presentation kept having lipase results within RI has not been previously reported and further supports the validity of our group allocation. It remains to be determined whether serum PAP-1 levels differ between AP and aGId when comparing dogs with both increased lipase and ultrasonographic evidence of AP (eg, enlarged and hypoechoic pancreas) to those with lipase within RI and a normal pancreas on ultrasonography. However, our results so far speak against this.

An important limitation of our study is the unequal group size, and post-hoc power analyses (see Supplementary file) suggest that some group comparisons should be interpreted cautiously. Nevertheless, given that our results are in line with those of a recent study demonstrating increased PAP-1 levels in dogs with aGId (based on single measurements),^27^ we consider our main conclusion to be robust.

Post-hoc analyses indicate that our study was well powered to detect within-dog changes over time in inflammation (CRP trajectories) but had limited power for some between-group contrasts, especially for PAP-1. Accordingly, non-significant PAP-1 group differences—and some CRP/PAP-1 contrasts—should be interpreted as inconclusive rather than evidence of no effect. Unequal group sizes further reduced power for between-group comparisons, and the inter-run imprecision of the PAP-1 assay means that only moderate-to-large between-run differences are reliably detectable in small cohorts. We therefore provide sample-size targets in the Supplement to achieve ~80% power for key contrasts using the observed effect sizes; these numbers are intended to guide future multicenter or staged studies (full procedure, estimates, and assumptions, see Supplementary data.)

When comparing our findings with other studies, it is important to consider that our simplified MCAI tends to yield slightly lower total scores. Nevertheless, when comparing gradings between our modified and the original MCAI,^12^ it becomes apparent that our categories were easier to assess and thus probably more reproducible in clinical practice than those of the original score.

In conclusion, serum PAP-1 did not differ statistically significantly between dogs with sAP and aGId. PAP-1 was significantly higher when some ultrasonographic features of AP were present. PAP-1 levels correlated significantly with clinical disease severity in aGId dogs, but not in sAP dogs. Significant correlations between PAP-1 and CRP in both groups suggest that serum PAP-1 levels reflect severity of inflammation in both pancreatic and intestinal tissues, and serum PAP-1 might serve as a potential biomarker of gastrointestinal inflammation in dogs.

## Supplementary Material

supplementary-material_aalag015

## Off-label antimicrobial declaration

Authors declare no off-label use of antimicrobials.

## Institutional animal care and use committee or other approval declaration

Approved by the cantonal veterinary office of Zurich.

## Appendix

### Evaluation of the SPARCL™ PAP-1 Immunoassay

PAP-1 concentration was measured at a dilution of 1:10 using the dog PAP-1 VetBio-1 assay and a VetBio-1 luminometer (Veterinary Biomarkers, Inc., West Chester, PA, USA). This assay uses Spatial Proximity Analyte Reagent Capture Luminescence (SPARCL™) technology, which is a proximity dependent, non-separation, chemiluminescent detection method. Dilution and measurement were performed according to the manufacturer’s instructions. In brief, 1:10 diluted sera were pipetted into 12 x 75 mm borosilicate tubes (Faust Laborbedarf AG, Schaffhausen, Switzerland), 0.5 mL of combined acridan and HRP conjugate was added. Tubes were gently mixed, incubated in the dark at room temperature (22.5°C) for 45 minutes and subsequently measured in the luminometer. With each sample series duplicates of 10 μL of diluent as zero standard and 10 μL of 0.400 μg/mL PAP-1 as standard (PAP-1-DOG-VB-S) were included. Based on the manufacturer’s recommendations, signals stronger than 1.5 x PAP-1 standard signal were considered potentially saturated; thus samples with higher concentrations are reported as > 6.0 μg/mL.

Linearity, intra- and inter-run precision, stability, and interferences were assessed. Measurement range was assessed by measuring two samples consisting of sera with high PAP-1 concentrations (6.13 and 5.32 μg/mL), and one sample with a lower PAP-1 concentrations (1.11 μg/mL). Serial two-fold dilutions were prepared, ranging from undiluted (1:1) to 1:32 (specifically 1:1; 1:2; 1:4; 1:8; 1:16 and 1:32). Each of these dilutions was subsequently diluted 1:10 using the standard assay buffer, and all dilutions were measured in triplicate. In addition, to specifically avoid potential matrix effects, two-fold serial dilutions were performed using PAP-1 standard (undiluted to 1:32, representing PAP-1 concentrations between 0.400 μg/mL down to 0.0125 μg/mL).

For intra-run precision, ten measurements of three pooled samples, one with a low PAP-1 concentration (reference serum, 0.43 μg/mL), one with a middle PAP-1 concentration (2.38 μg/mL) and the third with a higher PAP-1 concentration (4.58 μg/mL) were performed. Inter-run precision was assessed using a pooled sample with low PAP-1 and a pooled sample with high PAP-1 concentration stored at 22.5°C on five subsequent days, and additionally on day 8, and 15.

A spike recovery experiment was performed where PAP-1 standard was mixed with ten different ratios of a sample pool (0.135 μg/mL). Details on ratios and measurements can be found in the supporting excel file.

Influences of hemolysis, lipemia and icterus were determined by adding various concentrations of hemolysate (with hemoglobin concentrations of 5-1000 mg/dL as determined by Sysmex XN-1000), intralipid (Intralipid 20% Emulsion, I141-100ML; [Merck & Cie, Buchs Switzerland] resulting in a triglyceride concentration of 2.5–25.3 mmol/L as determined by Cobas), and bilirubin (Bilirubin ≥98%, powder, CAS: 635-65-4 (Merck & Cie), 2.5–30 mg/dL) to a pooled serum sample.

### SPARCL™ PAP-1 Immunoassay validation results

Linearity was assessed using two-fold serial dilutions ranging from 1:10 to 1:320 (6.13 μg/mL, and 1.11 μg/mL), and 1:10 to 1:160 (5.32 μg/mL), respectively. Results indicated excellent linearity (R^2^ of 0.9986 and 0.9915, respectively) at higher PAP-1 concentrations, and reduced linearity (R^2^ of 0.966) at lower PAP-1 concentrations. A lower limit of quantification of 0.2 μg/mL and upper limit of quantification (ULQ) of 6.0 μg/mL, which is in accordance with the information indicated by the manufacturer, was set. PAP-1 concentrations exceeding 6.0 μg/mL were reported as “> 6.0 μg/mL”, without specifying higher values.

PAP-1 concentrations were found to be in the range between 0.2 and > 6.0 μg/mL in patient samples. In total, twelve (18 %) and eight (6 %) PAP-1 values exceeded the ULQ of 6.0 μg/mL in the sAP and aGId group.

The coefficient of variation (CV) for intra-run precision was 3.8% (95% CI) for the sample with low PAP-1(0.43 μg/mL), 5.2% for the sample with middle PAP-1 (2.38 μg/mL) and 2.6% for the sample with higher PAP-1 concentrations (4.58 μg/mL). ins for inter-run precision were 22 % in the sample with low PAP-1 and 19 % in the sample with high PAP-1 concentrations. Storage of 15 days at room temperature led to a CV of 22% in a sample pool with low PAP-1 and 19% in a sample pool with high PAP-1 concentrations.

Spike-recovery experiments demonstrated good linearity across the tested dilution range, with an R^2^ of 0.9879 for the correlation between expected and measured values.

No interferences of hemolysis (6%), lipemia (3%), or hyperbilirubinemia (13%) were found. The right-sided RI for serum PAP-1 was 1.9 μg/mL (90% CI 1.2-2.2). All validation data can be found in the supporting excel file.

## Abbreviations

Agid: acute gastrointestinal disease
AP: acute pancreatitis
sAP: suspected acute pancreatitis
CRP: C-reactive protein concentration
DGGR: 1,2-o-dilauryl-rac-glycero-3-glutaric acid-(6 0-methylresorufin) ester
MCAI: modified canine activity index
UPASS: ultrasonographic pancreatic assessment severity score
ULQ: upper limit of quantification
