# Supplementary material for: Serum pancreatitis-associated protein 1 concentrations in dogs with acute signs of gastrointestinal disease and normal or abnormal DGGR lipase activity
Source: J Vet Intern Med. 2026 Feb 23;40(1):aalag015. doi: 10.1093/jvimsj/aalag015 (PMC12927877; doi:10.1093/jvimsj/aalag015)

Figure S1 Change in serum lipase activity over time in sAP dogs notable for consistently increased lipase activities above the inclusion threshold during hospitalization (n = 11). The gray-shaded area represents the RI. Please note that plotted points can be indistinguishable because of individual values that are too close or identical.


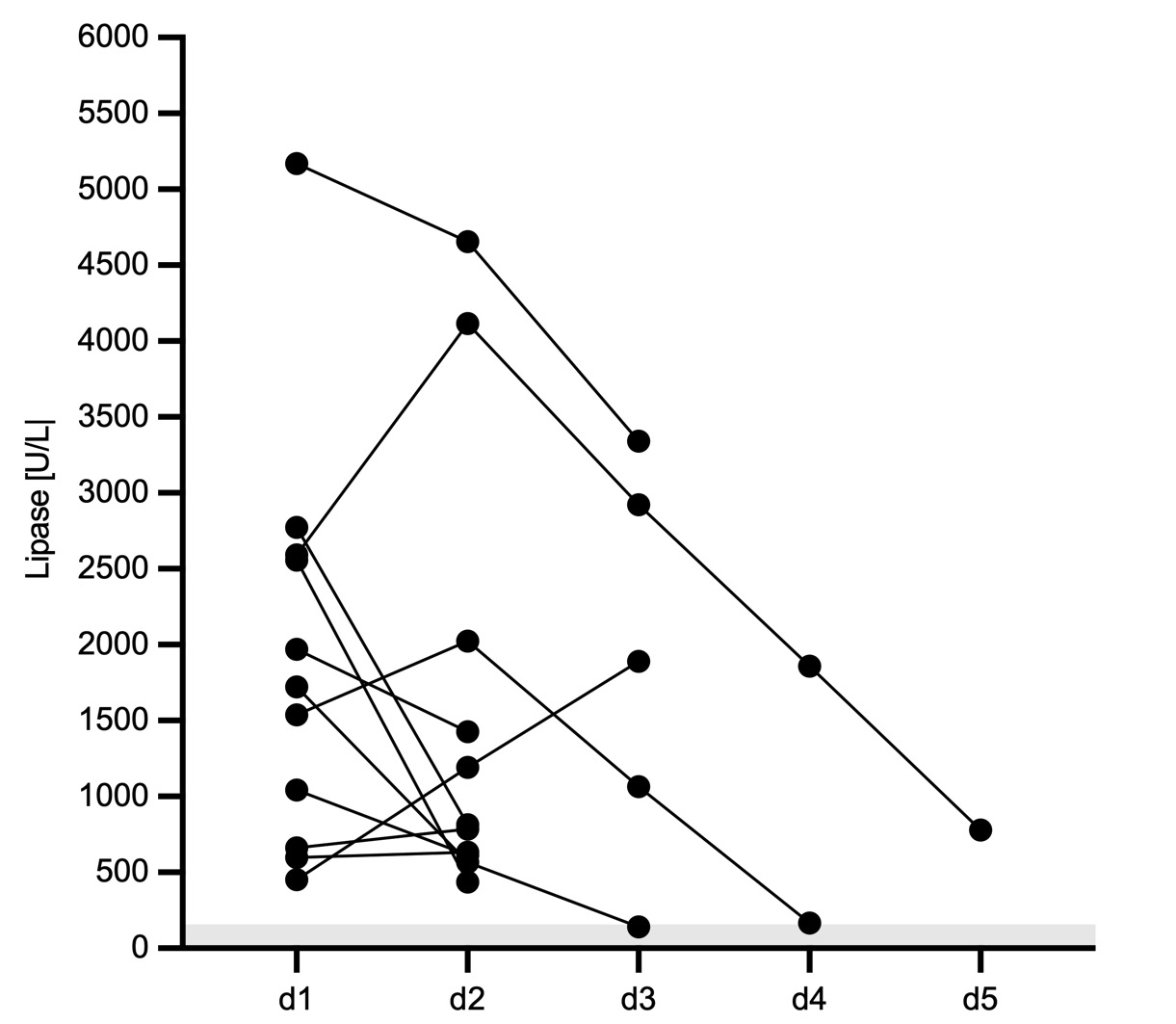


Figure S2 The ultrasonographic pancreatic severity score (UPASS) was significantly higher in sAP dogs notable for consistently increased lipase activities above the inclusion threshold during hospitalization (n = 11) compared to the rest of sAP dogs with a rapidly decreasing lipase activity (n = 15). Mann-Whitney U test, an alpha level of 0.05 was used to determine statistical significance.


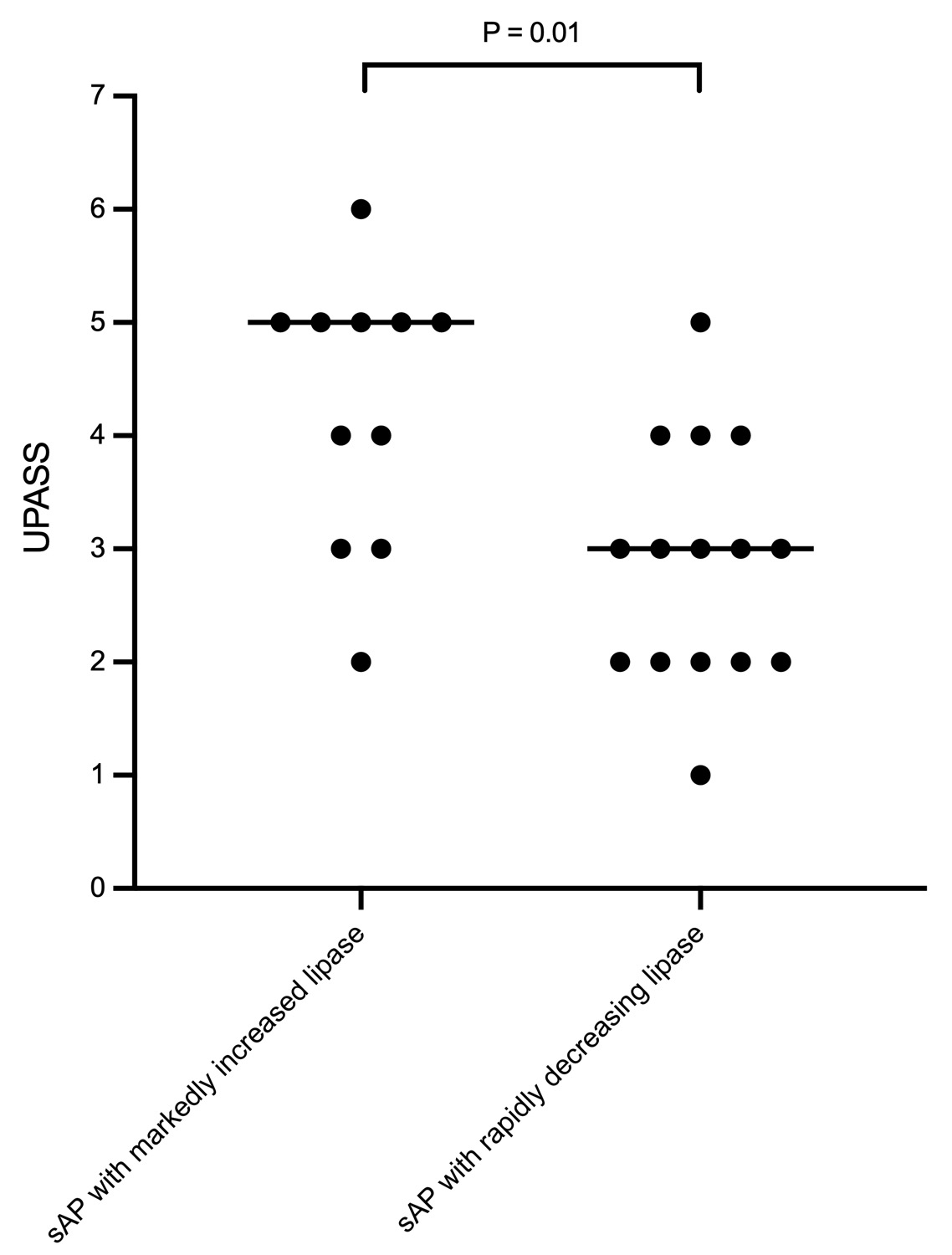


Figure S3 Changes in serum PAP-1 concentration over time in sAP dogs notable for consistently increased lipase activities above the inclusion threshold during hospitalization (n = 11). PAP-1 concentration at admission was significantly higher in these dogs compared to the rest of sAP dogs with a rapidly decreasing lipase activity (n = 15). Mann-Whitney U test, an alpha level of 0.05 was used to determine statistical significance. The gray-shaded area represents the RI. Please note that plotted points can be indistinguishable because of individual values that are too close or identical. This applies to dogs with PAP-1 values > 6.0 µg/mL in this Figure.


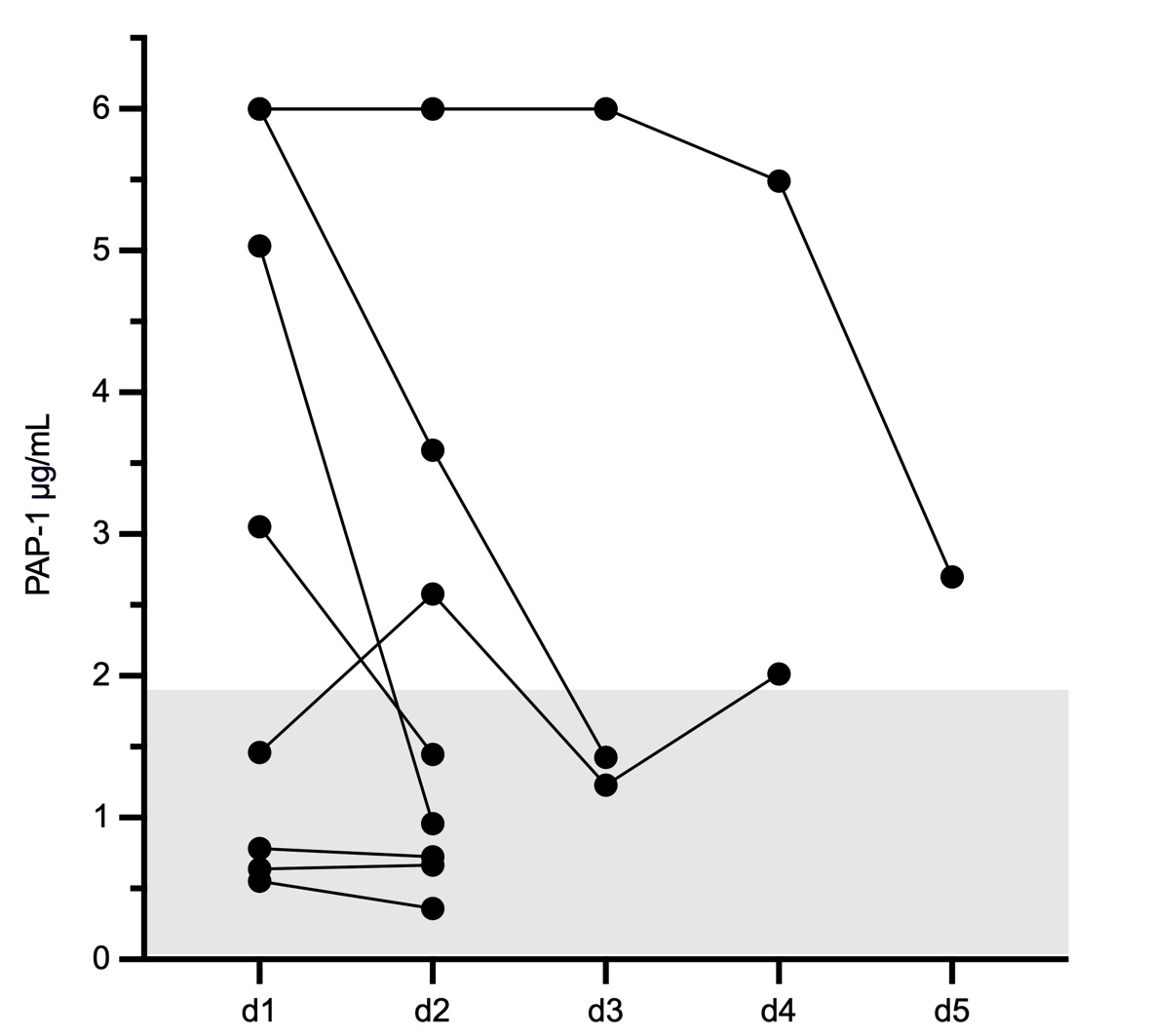


Figure S4 Change in serum CRP concentration over time in sAP dogs notable for consistently increased lipase activities above the inclusion threshold during hospitalization (n = 11). CRP concentration at admission was significantly higher in these dogs compared to the rest of AP dogs with a rapidly decreasing lipase activity (n = 15). Mann-Whitney U test, an alpha level of 0.05 was used to determine statistical significance. The gray-shaded area represents the RI. Please note that plotted points can be indistinguishable because of individual values that are too close or identical.


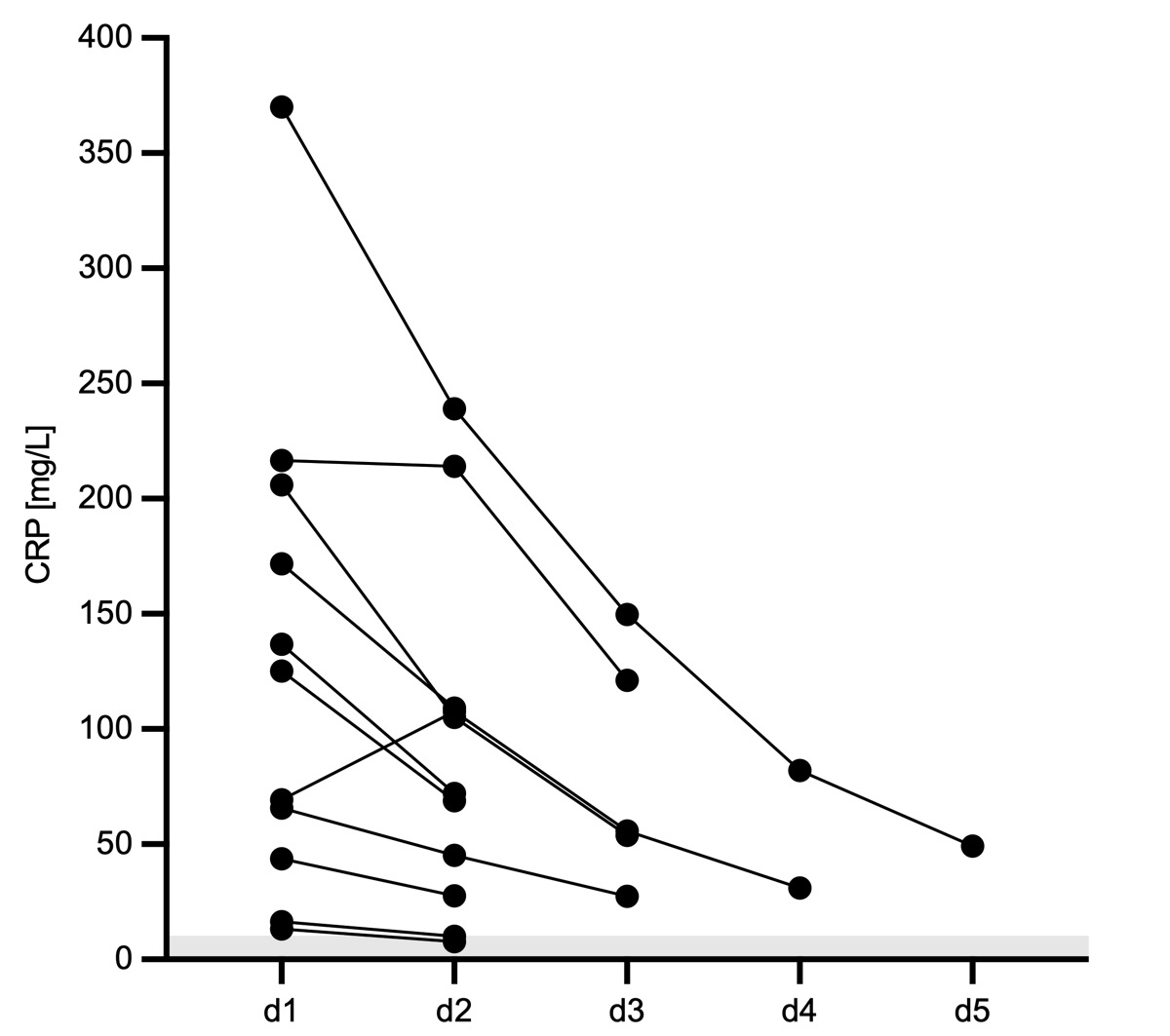

Supplement: supplementary-material_aalag015 [file supplementary-material_aalag015.zip › R3__Supplemental_files_Figures_S1-S3.docx]
