# Supplementary material for: Serum pancreatitis-associated protein 1 concentrations in dogs with acute signs of gastrointestinal disease and normal or abnormal DGGR lipase activity
Source: J Vet Intern Med. 2026 Feb 23;40(1):aalag015. doi: 10.1093/jvimsj/aalag015 (PMC12927877; doi:10.1093/jvimsj/aalag015)
Supplement: supplementary-material_aalag015 [file supplementary-material_aalag015.zip › Supplementary information_protocol Interferences.docx]

**PAP-1 paper: Protocols for the assessment of interferences**

**Preparation of Hemolysate and Hemoglobin-Spiked Plasma Samples**

Heparinized plasma was collected and washed twice with physiological saline (0.9% NaCl). After the final washing step, distilled water was added, and the sample was incubated overnight at −20 °C to induce lysis. Following thawing, the sample was mixed and centrifuged to remove cellular debris. Hemoglobin concentration in the resulting hemolysate was measured using a Sysmex XN-1000 analyzer and determined to be 13,200 mg/dL.

The hemolysate was subsequently diluted to achieve hemoglobin concentrations of 50, 100, 200, 400, 1,000, 2,000, 5,000, and 10,000 mg/dL. For each dilution, 25 μL of hemolysate was added to 225 μL of plasma, resulting in final hemoglobin concentrations of 5, 10, 20, 40, 100, 200, 500, and 1,000 mg/dL in the test samples. These concentrations represent a range from mild to marked hemolysis.

**Simulation of Lipemia**

To investigate the effects of lipids, lipemia was simulated by adding varying amounts of Intralipid (Intralipid 20% Emulsion, I242-100ML; Merck & Cie, Buchs, Switzerland) to aliquots of a pooled serum sample. Triglyceride concentrations in the resulting serum pools were measured using the Cobas analyzer and determined to be 25.3 mmol/L, 13.3 mmol/L, 7.0 mmol/L, 3.7 mmol/L, and 2.5 mmol/L. These concentrations represent a range from mild to marked lipemia.

**Effect of Hyperbilirubinemia**

To investigate the effects of hyperbilirubinemia, a bilirubin stock solution (10 mg/mL; ≥98% purity, powder, CAS: 635-65-4; Merck & Cie) was prepared in 0.1 M NaOH. This stock solution was added to a pooled plasma sample in a dilution series, resulting in final bilirubin concentrations of 2.5, 5, 10, 20, and 30 mg/dL. The resulting concentrations were confirmed by measurement using an automated chemistry analyzer (Cobas).
