# Supplementary material for: Serum pancreatitis-associated protein 1 concentrations in dogs with acute signs of gastrointestinal disease and normal or abnormal DGGR lipase activity
Source: J Vet Intern Med. 2026 Feb 23;40(1):aalag015. doi: 10.1093/jvimsj/aalag015 (PMC12927877; doi:10.1093/jvimsj/aalag015)
Supplement: supplementary-material_aalag015 [file supplementary-material_aalag015.zip › Supplementary_posthoc_results_including_table_1_Posthoc_Power_revDB.docx]

Supplementary file on details of post-hoc power anaylses

M&M

A priori power analysis to estimate the minimum required sample size for detecting PAP-1 group and time effects could not be conducted due to the absence of data at the study’s outset. One of the aims of this study was to generate an initial dataset that would enable such analyses in future research. Consequently, we performed a post hoc power analysis for the PAP-1 group and time effects, as well as for lipase activity and CRP, to assess the statistical power achieved with our dataset. Additionally, we estimated the sample sizes that would have been necessary to reach the conventional threshold of 80% power (1 − β), in cases where the observed power fell below this benchmark. Post hoc power was evaluated for the fixed effects of group and time using the generalized linear mixed model (GLMM) described above, implemented in R 4.4.0 with the glmmTMB package (version 1.2.4). From the fitted model, we extracted fixed-effect coefficients, variance components, and covariance parameters, which were treated as the data-generating mechanism for the power simulations. For each biomarker (lipase, CRP, and PAP-1), we generated 1,000 parametric bootstrap datasets that preserved the original sample size, time schedule, and missing data structure. Each simulated dataset was re-analyzed using the same GLMM, and Wald χ² tests were conducted for the group and time effects. Observed power was calculated as the proportion of bootstrap p-values < 0.05 (two-sided). Monte Carlo 95% confidence intervals (CIs) were derived using the exact binomial distribution. The choice of 1,000 bootstrap replicates reflects a balance between computational efficiency and the precision of power estimates, which is generally considered sufficient for stable inference in simulation-based analyses.

*Results - Post-hoc power analysis of lipase activity, CRP, and PAP-1 change over time*

Supplementary Table 1 shows the achieved power for all fixed effects. In brief, assuming a traditional power threshold of 80% for in vivo observations, the study was decisively powered for the group effect on lipase activity (rate ratio [RR] = 3.20; power = 0.99, 95 % CI 0.98–1.00) and for its time trend (0.88; RR = 1.15 day⁻¹, 95 % Cl 0.85-0.91). For CRP, power was adequate for the time effect (0.83; RR = 1.11 day⁻¹, 95 % Cl 0.80-0.86) but low for the group contrast (0.28; RR = 1.21, 95 % Cl 0.25-0.32). PAP‑1 displayed moderate power for the time effect (0.62; RR = 1.05 day⁻¹, 95 % Cl 0.58-0.66) and low power for the group effect (0.34; RR = 1.32, 95 % Cl 0.30-0.38). Confidence limits around all estimates were narrow. Because balanced allocation maximizes power, unequal groups require more total dogs to reach the anticipated minimum power and may have contributed to certain low to moderate power estimations. Retaining the present group allocation ratio of dogs and assuming the estimated effects sizes hold true in future studies, the minimum (rounded) animal numbers to achieve 80 % power for the group effects of CRP and PAP-1 as well as the time effect of PAP-1 are: CRP – group effect:  99 dogs in group 1 and 54 dogs in group 2 (total 153). PAP‑1 – group effect: 85 dogs in group 1 and 47 dogs in group 2 (total 132). PAP‑1 – time effect: 52 dogs in group 1 and 29 dogs in group 2 (total  81).

## Supplementary Table 1. Post‑hoc power estimation for fixed effects on serum lipase, CRP, and PAP-I assuming a generalized repeated measures mixed model (group, time) with a gamma error distribution and log-link function^1^.

| Response | Effect | Effect size^2^ | Observed power | 95 % CI |
| --- | --- | --- | --- | --- |
| Lipase | Group | 3.20 RR | 0.99 | 0.98 – 1.00 |
| Lipase | Time | 1.15 RR /day | 0.88 | 0.85 – 0.91 |
| CRP | Group | 1.21 RR | 0.28 | 0.25 – 0.32 |
| CRP | Time | 1.11 RR /day | 0.83 | 0.80 – 0.86 |
| PAP‑I | Group | 1.32 RR | 0.34 | 0.30 – 0.38 |
| PAP‑I | Time | 1.05 RR /day | 0.62 | 0.58 – 0.66 |

^1^Repeated measures GLIMMIX included the animal as a random factor and assumed a ANTE(1) covariance structure. Model fit was assessed with the Bayesian Information criterion.

^2^RR = rate ratio, calculated as exp(β); exp denotes the natural exponential function and β is the fixed‑effect coefficient on the log scale. CI = Monte‑Carlo 95 % confidence interval. Sample sizes: group 1 = 49 animals, group 2 = 27 animals. Power estimates are based on 1 000 parametric bootstrap replicates per biomarker.
